# The acquired *pco* gene cluster in *Salmonella enterica* mediates resistance to copper

**DOI:** 10.3389/fmicb.2024.1454763

**Published:** 2024-09-03

**Authors:** Ahmed F. Hikal, Sameer Hasan, Dereje Gudeta, Shaohua Zhao, Steven Foley, Ashraf A. Khan

**Affiliations:** ^1^Division of Microbiology, National Center for Toxicological Research, United States Food and Drug Administration, Jefferson, AR, United States; ^2^Office of Applied Science, Center for Veterinary Medicine, U.S. Food and Drug Administration, Laurel, MD, United States

**Keywords:** copper resistance, *pco* cluster, *Salmonella enterica*, heavy metal resistance, *pcoABCD genes*

## Abstract

The pervasive environmental metal contamination has led to selection of heavy-metal resistance genes in bacteria. The *pco* and *sil* clusters are located on a mobile genetic element and linked to heavy-metal resistance. These clusters have been found in *Salmonella enterica* serovars isolated from human clinical cases and foods of animal origin. This may be due to the use of heavy metals, such as copper, in animal feed for their antimicrobial and growth promotion properties. The *sil* cluster can be found alone or in combination with *pco* cluster, either in the chromosome or on a plasmid. Previous reports have indicated that *sil*, but not *pco,* cluster contributes to copper resistance in *S. enterica* Typhimurium. However, the role of the *pco* cluster on the physiology of non-typhoidal *S. enterica* remains poorly understood. To understand the function of the *pco* gene cluster, a deletion mutant of *pcoABCD* genes was constructed using allelic exchange mutagenesis. Deletion of *pcoABCD* genes inhibited growth of *S. enterica* in high-copper medium, but only under anaerobic environment. Complementation of the mutant reversed the growth phenotype. The survival of *S. enterica* in RAW264.7 macrophages was not affected by the loss of *pcoABCD* genes. This study indicates that the acquired *pco* cluster is crucial for copper detoxification in *S. enterica*, but it is not essential for intracellular replication within macrophages.

## Introduction

*Salmonella enterica* serovars are among the most common causes of bacterial gastrointestinal disease worldwide and pose a major public health concern with an estimated 1.35 million *Salmonella* infections including 26,000 leading to hospitalization, and 420 deaths each year in the United States (European Food Safety Authority and European Centre for Disease Prevention and Control, 2019; Centers for Disease Control and Prevention, 2023). Globally, it is estimated that over 93 million cases of *Salmonella* gastroenteritis and 155,000 deaths occur annually, leading to the exacerbation of the global burden of Salmonellosis (Majowicz et al., 2010). Antimicrobial resistance in *S. enterica* is a public health concern due to the rapid spread of the multidrug-resistant (MDR) strains, which is linked to the overuse of antibiotics (Punchihewage-Don et al., 2022). These MDR strains cause approximately 410,000 infection every year in the U.S. (Punchihewage-Don et al., 2022).

Horizontal gene transfer mediates antimicrobial resistance among different bacterial species through the exchange of mobile genetic cassettes, which are often on transferrable plasmids or transposons (Sun et al., 2019). Notably, natural transfer of conjugative plasmids is initiated when bacteria are exposed to environmental stresses, which can induce the DNA uptake system of the recipient cell (Sun et al., 2019). Non-typhoidal *Salmonella* have acquired heavy-metal resistance genes for copper (Cu) and silver (Ag) to cope with the high level of heavy metals pollutants in the environment and this metal tolerance was found to be co-occur with antibiotic resistance (Mourao et al., 2016). MDR cassettes that carry antimicrobial resistance genes are commonly associated with heavy-metal resistance genes, suggesting that co-selection of antimicrobial resistance can occur in the presence of heavy metals, even at sub-inhibitory concentrations of these metals. The *pco* and *sil* clusters have also been detected in association with the arsenic (*ars*) resistance genes in the epidemic clone of *S.* Typhimurium, suggesting that these clones can survive in environments with various types of heavy-metal contaminants (Joana Mourao et al., 2020; Branchu et al., 2019). More recently, a whole genome sequence (WGS) analysis using long-read sequencing of 134 MDR *S. enterica* isolated from retail meat revealed that 60% of these MDR strains carry heavy-metal resistance genes along with AMR genes, which raise the concern of co-selection of heavy metal and antibiotic resistance (Li et al., 2021).

Copper is an essential trace element for all bacteria. The redox-active property of copper is crucial for cuproenzymes, such as the cytochrome c oxidase respiratory super complexes and superoxide dismutase, which protect bacteria from superoxide radicals generated by macrophages and neutrophils as a defense mechanism against invading pathogen (Samanovic et al., 2012). However, excess copper is detrimental to cells as it replaces native metal cofactors in proteins leading to their damage. Therefore, in response to copper, bacteria have evolved sophisticated mechanisms to evade copper toxicity either by extruding or sequestering copper, or by oxidizing the toxic Cu (I) to less toxic Cu (II) (Giachino and Waldron, 2020). For example, *S. enterica* serovar Typhimurium encodes CopA and GolT, metal-transporting P-type ATPase proteins, which export copper outside the cell to maintain copper homeostasis (Osman et al., 2010). In addition to *copA* and *golT*, *S.* Typhimurium carries the gene that encodes CueO, a periplasmic multicopper oxidase, which oxidizes Cu (I) to Cu (II); deletion of *cueO* gene resulted in severe attenuation in mouse model (Achard MET et al., 2010). Essential trace metals, such as copper, zinc, molybdenum, selenium, manganese, iron, and cobalt are extensively used in animal and poultry feed as nutritional additives to promote growth (Hejna et al., 2018). The recommended copper concentration in poultry feed by the National Research Council (NRC) is 8 mg/kg (Subcommittee on Poultry Nutrition CoAN, 1994), whereas the maximum concentration of copper allowed in poultry and animal feed by the European Union (EU) is ranging from 15–35 mg/kg, except in piglets (up to 12 weeks) feed, where 170 mg/kg can be permitted (EFSA Panel on Additives and Products or Substances used in Animal Feed, 2016). But in fact, copper is over-supplemented in animals diet (up to 800 mg/kg) (Korish and Attia, 2020). Despite its insidious toxicity, copper was found to be over supplemented in animals feed compared to other trace metals (Lopez-Alonso and Miranda, 2020). Consequently, this overuse has potentially led to increase the minimal inhibitory concentration (MIC) by opportunistic bacteria such as *Enterococcus* spp. to high levels of copper, which is associated with the acquisition of copper-resistance genes (Yazdankhah et al., 2014).

In *Escherichia coli*, the *pco* cluster contains seven genes, *pcoABCDERS* (Brown et al., 1995). *pcoA* encodes the multicopper oxidase, PcoA, which oxidizes Cu (I) to the less toxic Cu (II) form in the periplasm; whereas, PcoC is a periplasmic protein that binds one ion of Cu (II) (Huffman et al., 2002). PcoE is also a periplasmic protein, which is thought to act as a “metal sponge” that scavenges free Cu ions from the periplasmic space (Zimmermann et al., 2012). On the other hand, PcoB is an outer membrane protein and predicted to act as a Cu (II) importer that may facilitate Cu uptake in Cu-scarce environment or sequester excess Cu (Li et al., 2022). PcoD is a predicted inner membrane protein of unknown function. The *pco* and *sil* gene clusters (frequently found in *Enterobacteriaceae* species) are co-transferred from one bacterium to another by a Tn7-like transposon element (32.54 kb), which is located between the integrons (Fang et al., 2016; Billman-Jacobe et al., 2018; Lee et al., 2002). Additionally, two studies have shown that the *sil* gene cassette alone, without the *pco* cluster, conferred resistance to high levels of Cu under anaerobic conditions (Mourao et al., 2016; Mourao et al., 2015). These results suggest that *sil* genes can handle copper toxicity without the need of the *pco* cassette. The *pco* and *sil* clusters were recently detected in the *Salmonella* genomic island 4 (SGI-4), an integrative conjugative element (ICE), in monophasic *S.* Typhimurium; only deletion of *silABC,* but not *pcoABC*, reduced the minimum inhibitory concentration (MIC) to CuSO_4_ under anaerobic conditions (Branchu et al., 2019). A similar study also found the *cus* (renamed to *sil*) and *pco* clusters in SGI-3 in *S.* Typhimurium; the loss of *pco* cluster did not result in growth defects in copper-supplemented medium under anaerobic environment (Arai et al., 2019). However, the role of *pco* cluster in tolerance to toxic levels of Cu (I) is still unclear.

The present study examines the role of the *pco* gene cluster in non-typhoidal *Salmonella* for copper resistance and survival in macrophages. Our work shows that the acquired *pco* cluster contributes to Cu (I) resistance. However, our study reveals that the acquisition of the *pco* cluster has no impact on replication efficiency within macrophages.

## Materials and methods

### Bacterial strains

The *S. enterica* isolates used in this study were isolated from retail meat in the United States (Table 1) (Li et al., 2021; McDermott et al., 2016). Growth phenotype was compared to the wild-type *S. enterica* ATCC13076 (lacks *pco* and *sil* clusters). Analyzing the whole genome sequence of SL-3 and SL-4 and identifying the location of the *pco* and *sil* cluster was performed using the MOB-RECON software tool in the cloud-based Galaxy bioinformatics platform.^1^

**Table 1:** Bacterial strains.

Strain	Serovar	Designation	Trace element resistance genotype	Accession number
N31410	Anatum	SL-3	*pcoABCDERSE1E2G – silPABCRSE*	GCA_001479145.1
N32755	Senftenberg	SL-4	*pcoABCDERSE1E2G – silPABCRSE*	GCA_001479465.1
N18S0981	Typhimurium	SL-27	*silPABCRSE*	GCA_007861645.2
ATCC13076	Enteritidis	WT	__	GCA_001643395.1

### Construction of a *pco* deletion mutant and complementation

To create mutation in the *pco* cassette, the upstream region of *pcoA* was amplified from SL-4 with primers: P27 and P28 (Table 2), and the downstream region of *pcoD* was amplified with primers: P29 and P30 (Table 2) to create overlapping PCR products. The upstream and downstream PCR products of *pcoA* and *pcoD* were cut with restriction enzymes: *Sal*I and *Sac*I, respectively and inserted into the *Sal*I and *Sac*I sites of the temperature-sensitive plasmid pDG3 (encodes β-galactosidase gene, *bgaB,* for blue/white screening) (Gudeta and Foley, 2024) to generate plasmid pAH3 using NEBuilder HiFi DNA assembly kit (New England BioLabs, Ipswich, MA, USA). SL-4 was then electroporated with plasmid pAH3. To allow integration of pAH3 in SL-4 chromosome, transformants were plated onto LB agar supplemented with 0.5% glucose, X-Gal 80 μg/mL, and 25 μg/mL chloramphenicol and incubated at 42°C overnight. A single blue colony from the transformants plate was inoculated in LB broth supplemented with 1% rhamnose and incubated at 30°C for 4 h. To facilitate allelic replacement of *pcoABCD* with the upstream and downstream PCR products on pAH3, cells were then harvested by centrifugation at 10,000 × *g*, resuspended in LB broth, plated onto LB agar plates without antibiotics, and incubated at 37°C for 24 h for blue/white screening. White colonies were screened to confirm the loss of *pcoD* using primers P19 and P24. To complement SL-4Δ*pcoABCD* mutant, we amplified *pcoEABCDRS* genes from the parent strain using primers: P55 and P56, which contain *Sal*I and *Sac*I restriction enzyme sites, respectively. The *pcoEABCDRS* PCR product was cut with *Sal*I and *Sac*I restriction enzymes and inserted into the *Sal*I and *Sac*I sites of plasmid pBBR1-MCS2 to create pAH8 (*SI* 1A). Then, pAH8 was electroporated into SL-4Δ*pcoABCD*. To facilitate the delivery of the 12 kb pAH8 in SL-4Δ*pcoABCD*, cells were grown in LB supplemented with 8 μg/mL polymyxin B nonapeptide (permeabilizes bacterial outer membrane) (Qin et al., 2022) and 50 μg/mL kanamycin before electroporation. Transformants were plated onto LB agar plates supplemented with 50 μg/mL kanamycin and 3 mM CuSO_4_ and incubated at 37°C anaerobically for 3 days. The complemented SL-4Δ*pcoABCD* was verified by PCR using primer pairs: P23/P56 and P61/P24.

**Table 2:** Primers used in the study.

Primer	Sequence (5’ → 3)
P19	TCATCACATTCTCCGATGCC
P23	ATCGCCGATATGACCGTTG
P24	GCAACTCAAGCAGAACGTATTC
P27	GGGCGCGCGCCATTCTCCGGTCGACTTTGTAACATATCTTGTTTCCCGTTTGC
P28	GAGCTTTGGATGTATGAATATATTAATCACGACCACTGCG
P29	TAATATATTCATACATCCAAAGCTCATGCGTG
P30	ATAGGCGCGCCACTCGAGGAGCTCTCACATTCGTATCACTGTCAAAATTCATG
P55	GTCACAGTCGACGCACCTGACGCTAAGCACTAAC
P56	CAGTCAGAGCTCCATTTTTGTCACCTTCCGGGTTTCTC
P61	GGAATTAAAAAGCTCTGTGCCACAGG

### Growth conditions and *in vitro* growth curves

Strains were grown in LB only or LB supplemented with 4 mM CuSO_4_ or LB agar supplemented with 2 mM CuSO_4_. For the copper-tolerance growth curves, overnight cultures of *S. enterica* isolates grown in LB broth at 37°C. Kanamycin (50 μg/mL) was added to SL-4Δ*pcoABCD/*empty vector (pBBR1-MCS2) and the complemented strain to maintain the pBBR1-MCS2 and pAH8, respectively. Bacterial cultures were diluted to an OD_600_ of 0.05 in the same medium and inoculated in 96-well plates containing LB broth only or LB broth supplemented with 3 mM CuSO_4_. The cultures were incubated stationary at 37°C under anaerobic condition for 7 days. Anaerobic atmosphere was generated using Mitsubishi AnaeroPack-Anaero (Thermo Fisher Scientific, Waltham, MA, USA). Cultures were mixed immediately before measuring the optical density (OD_600_) at the designated time points.

### Minimum inhibitory concentrations (MICs) to copper sulfate

Minimum inhibitory concentrations of *S. enterica* strains to CuSO_4_ were determined by the agar dilution assay. Strains were inoculated from a single colony into LB broth and incubated at 37°C overnight then diluted to approximately 10^−7^ CFU/mL and 5 μL spotted onto Muller-Hinton agar supplemented with CuSO_4_ (0.5, 0.75, 1, 1.5, 2, 3, 4, 5, 6, 7, 8, and 12 mM). Plates were incubated aerobically or anaerobically at 37°C for 24 or 72 h, respectively.

### Alamar blue assay

Strains of *S. enterica* were inoculated to OD_600_ of 0.05 into 96-well plates containing LB broth alone or LB supplemented with 1–3 mM CuSO_4_ and incubated at 37°C aerobically or anaerobically overnight or 72 h, respectively. Next, 20 μL alamarBlue reagent (Thermo Fisher Scientific, Waltham, MA, USA) was added to each well, and plates were incubated for 4 h at 37°C. The assessment of viable bacteria was according to the reduction of the blue indicator, resazurin to resorufin (pink).

### Macrophage infection

RAW264.7 macrophage cell lines were cultured in DMEM basal medium supplemented with 10% fetal bovine serum (DMEM-FBS) at 37°C and 5% CO_2_ and replaced daily with fresh pre-warmed medium. Cells were seeded in 24-well plates at a density of 1.5 ×10^6^ cells per well and incubated at 37°C and 5% CO_2_ until reached confluency. RAW264.7 macrophage were then infected with *S. enterica*: WT, SL-3, SL-4, and SL-4Δ*pcoABCD* strains at an MOI of 1 (1 bacteria/macrophage cell) for 1 h at 37°C. At indicated time-points, cells were washed twice with phosphate buffered saline (PBS) and lysed with 1% triton X-100 for 10 min. Cell lysates were serially diluted in PBS, plated onto LB agar plates, and incubated for 24 h at 37°C for colony enumeration.

### Statistical analysis

Statistical analyses of bacterial growth data were performed using Student’s unpaired *t* test (two-tailed). Statistically significant difference in growth curves and replication in macrophages between the groups was calculated using ANOVA with pair-wise comparisons.

## Results

### *pco* cluster structure and location

**Figure 1:**
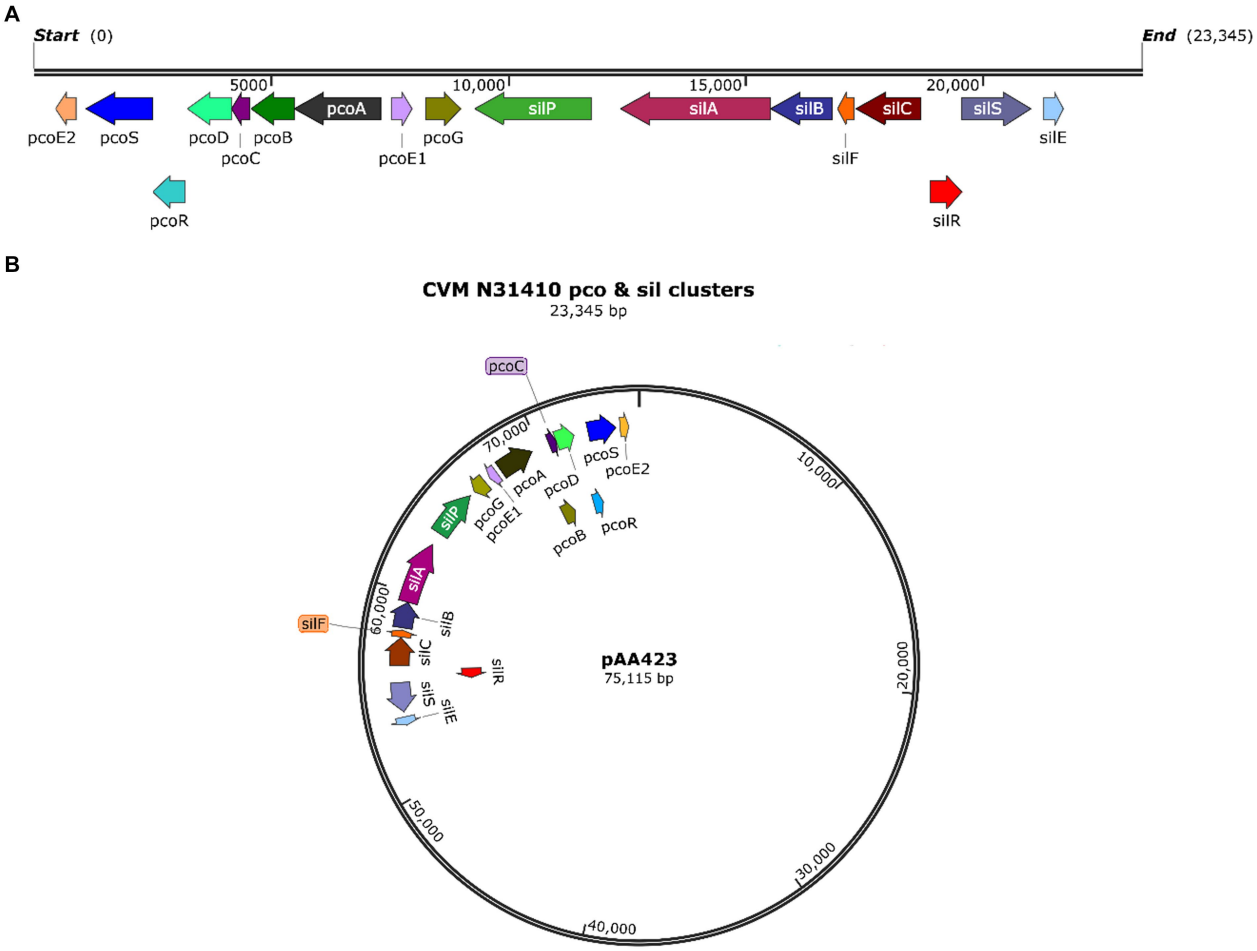
**(A)** Map of the *pco* and *sil* gene cassette in the chromosome of *S. enterica* CVM N31410 (SL-3) isolate. **(B)** Plasmid map of the pAA423 plasmid located in *S. enterica* CVM N32755 (SL-4) isolate. The maps were created by SnapGene 7.0. 2.

**Figure 2:**
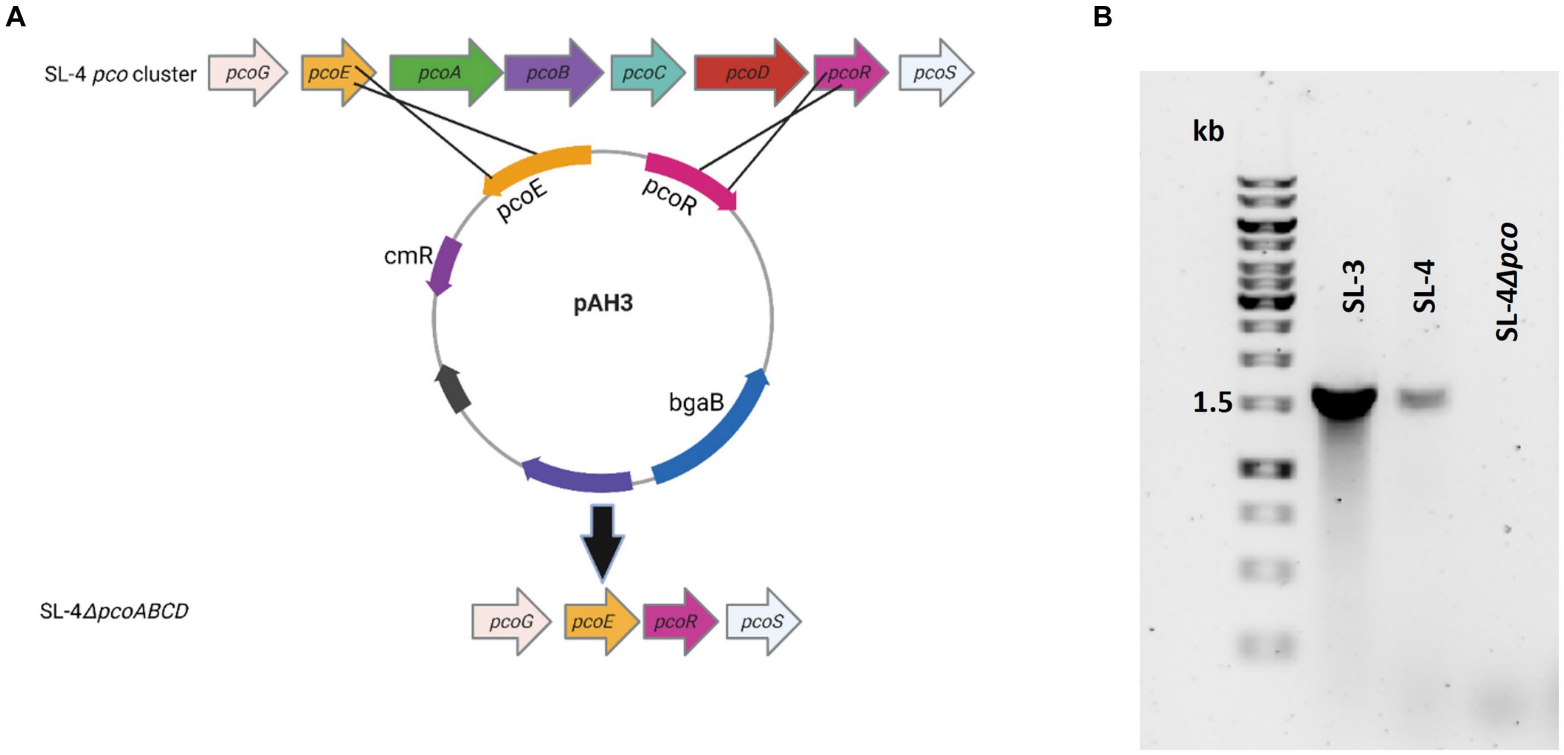
Confirmation of *pcoABCD* mutation. **(A)** Overview of SL-4 *pcoA-D* deletion. Plasmid pAH3 encompasses the upstream (*pcoE*) and downstream region (*pcoR*) regions of *pcoA-D* was used to replace *pcoA-D*. **(B)** PCR screening for the loss of *pcoD* using primers: P-19 and P-24. The expected amplicon size for the presence of the gene is 1.6 kb.

*In silico* analysis of the whole genome sequence showed that *S. enterica* serovar Anatum isolate N31410 (SL-3) and *S. enterica* serovar Senftenberg isolate N32755 (SL-4) contain the *pcoABCDERSE1E2G* genes; these genes are located adjacent to silver-resistance cassette, *silPABCRSE* (Figure 1A). The *pco* and *sil* clusters are located in the chromosome of *S. enterica* serovar Anatum isolate N31410 (SL-3) (Figure 2A). Intriguingly, *S. enterica* serovar Senftenberg isolate N32755 (SL-4) carries *pco* and *sil* genes cluster on a 75 kb incompatibility group (Inc) FIB plasmid (AA423) (Figure 1B), which is unusual for those genes to occur on plasmids of the IncF family. Remarkably, these gene clusters are not located in conjunction with or in the vicinity of antimicrobial-resistant (AMR) genetic elements, such as *blaTEM-1*, *aadA*, *aac(6″)-Ib4*, *sul*, and *tetA* as they are frequently co-located with these antibiotic resistance genes (Mourao et al., 2016).

### The *pco* genes cluster is required for copper tolerance

**Figure 3:**
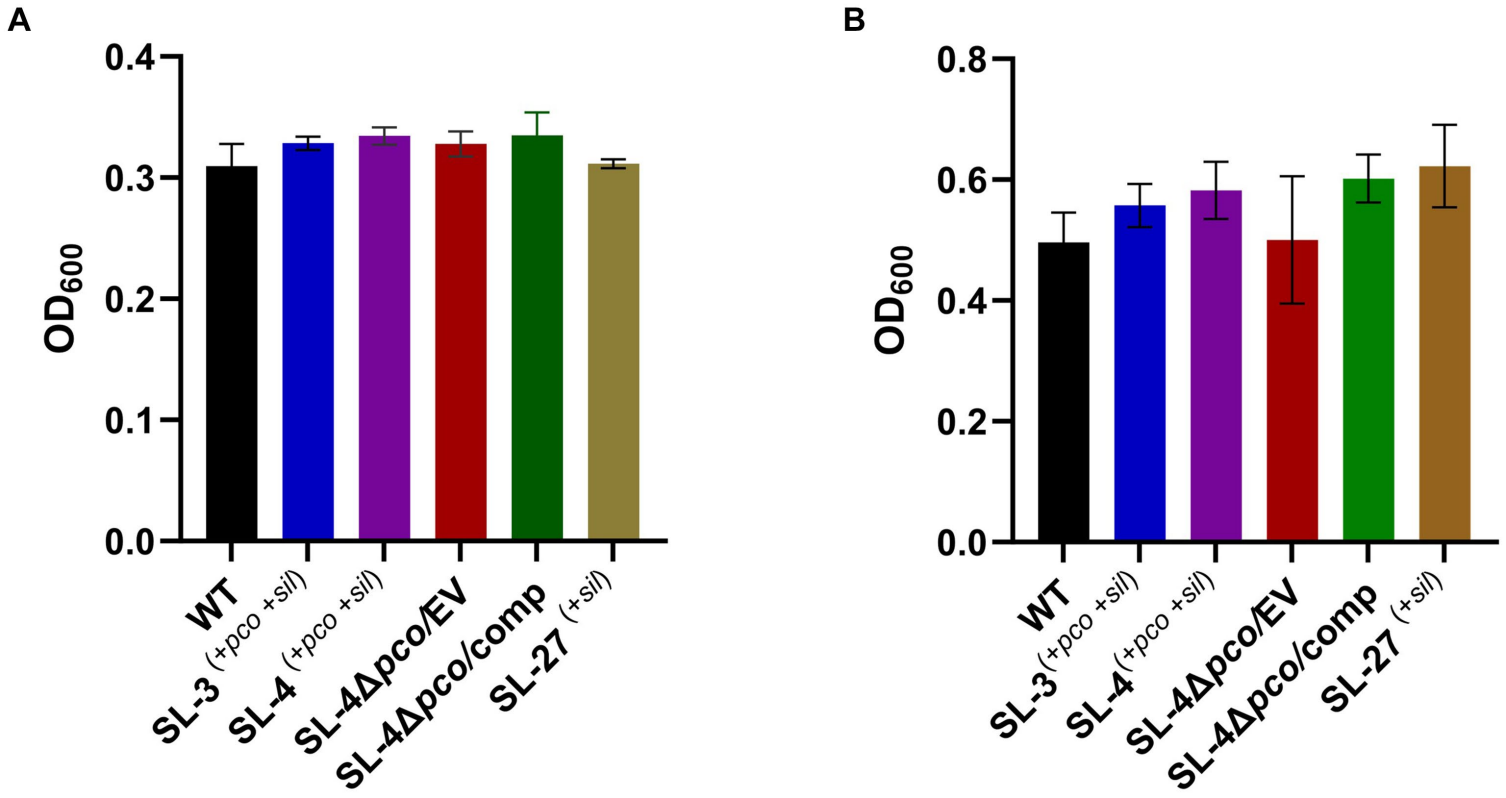
Assays for the effect of *pcoABCD* deletion on growth in LB supplemented with CuSO_4_ or LB only under aerobic or anaerobic conditions, respectively. WT *S. enterica*, SL-3, SL-4, SL-4Δ*pco/*EV (empty vector), SL-4Δ*pco/*comp (complement), and SL-27 strains were cultured in LB with 4 mM CuSO_4_ and incubated aerobically for 24 h **(A)** or grown in LB only and incubated anaerobically **(B)**. Error bars represent the standard error from two independent experiments in triplicates. The *p-*values were calculated by unpaired two-tailed Student’s *t* test. All *p-values* are greater than 0.05.

**Figure 4:**
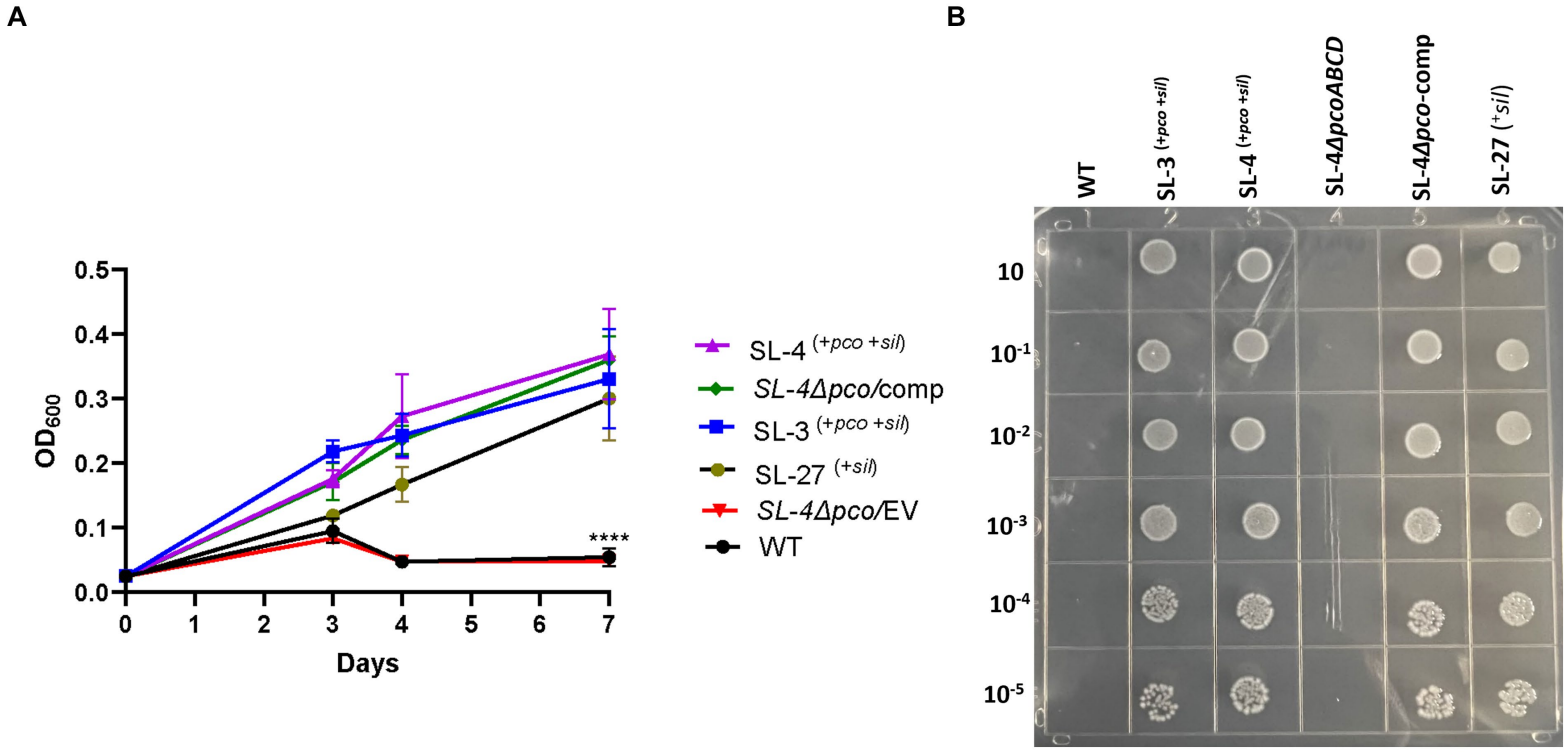
The *pco* cluster enhances tolerance to high levels of copper under anaerobic conditions. **(A)** Growth curve of WT *S. enterica*, SL-3, SL-4, SL-4Δ*pco/*EV (empty vector), SL-4Δ*pco/*comp (complement), and SL-27 in LB broth supplemented with 3 mM CuSO_4_. Cultures were incubated at 37°C anaerobically for 7 days. Cell densities shown (OD_600_) are the mean +/− standard error from three independent cultures assayed in triplicates. The *p-*values were calculated by two-way ANOVA (***p ≤ 0.0001). **(B)** Plating results of the same strains that were grown in LB broth then serially diluted spotted onto LB agar supplemented with 2 mM CuSO_4_. Plates were then incubated at 37°C anaerobically for 72 h.

To assess roles of the *pco* genes cluster in copper tolerance, we created a deletion of *pcoA-D* cassette in SL-4 through allelic exchange mutagenesis (Figure 2A). The deletion mutant was confirmed by PCR (Figure 2B). Growth of strains was tested in LB medium only or LB medium containing high levels of copper. In this experiment, we tested copper resistance of the parent strain SL-4 and SL-3. Additionally, we compared growth rate of SL-3, SL-4, and SL-4Δ*pcoABCD,* and SL-4Δ*pcoABCD*-complement to *S. enterica* Typhimurium (SL-27), which carries *sil* cluster only, and *S. enterica* ATCC 13076 (negative control). All strains were grown in LB supplemented with 3 mM CuSO_4_. Bacterial cultures were incubated aerobically or anaerobically with or without CuSO_4_. The results show that there is no significant difference in growth between all the strains growing in copper-supplemented medium under aerobic condition (Figure 3A). Also, all the strains grow at the same level in LB broth only under aerobic (data not shown) or anaerobic condition (Figure 3B). Because bacterial growth rate slows down in anaerobic environment, and we also wanted to examine if Cu (I)-resistant *S. enterica* can tolerate long-term exposure of high levels of copper, we monitored growth of anaerobic culture for 7 days. Our data reveal that deletion of the *pcoABCD* cassette resulted in a significant growth reduction in Cu-supplemented medium when incubated anaerobically (Figure 4A). Growth was restored by the complemented SL-4Δ*pcoABCD* strain (Figure 4A). SL-3^(+*pco* + *sil*)^, SL-4^(+*pco* + *sil*)^, and SL-27^(+*sil*)^ exhibited high resistance to copper and grew at a significantly higher growth rate than the mutant and wild-type *S. enterica* (Figure 4A). We also examined the MICs of *S. enterica* strains to copper on solid medium (Table 3). The MIC of all strains to CuSO_4_ was the same (8 mM) under aerobic condition. Under anaerobic environment, the MIC of SL-3^(+*pco* + *sil*)^, SL-4^(+*pco* + *sil*)^, SL-4Δ*pcoABCD*-complement, and SL-27^(+*sil*)^ was 7 mM, whereas the MIC of SL-4Δ*pcoABCD* and WT was 1 mM, where no visible colonies were formed (Figure 4B). We did not see any difference in the MIC between the strains carrying both *pco* and *sil* clusters (SL-3 and SL-4) and SL-27, which harbors *sil* cassette only.

**Table 3:** MICs to CuSO_4_.

Strain	MIC (mM)	
	Aerobic	Anaerobic
WT-ATCC 13076	8	1
SL-3^(+*pco* + *sil*)^	8	7
SL-4^(+*pco* + *sil*)^	8	7
SL-4Δ*pcoABCD*	8	1
SL-4Δ*pcoABCD-*complement	8	7
SL-27^(+*sil*)^	8	7

Growth inhibition of the *pco* mutant by copper in anaerobic environment was also confirmed by Alamar Blue assay. Growth of the SL-4Δ*pcoABCD* mutant in Cu-supplemented medium was not impacted when incubated aerobically (Supplementary Figure S2A). However, the mutated exhibited growth inhibition (blue color) in LB supplemented with CuSO_4_ (1–3 mM) when compared with the parent strain and SL-3 cultures (pink color) (Supplementary Figure S2B).

Together, these data suggest that *pcoABCD* genes contribute to copper (I) tolerance in *S. enterica*.

### Loss of the *pcoABCD* genes does not have an impact on replication in macrophages

**Figure 5:**
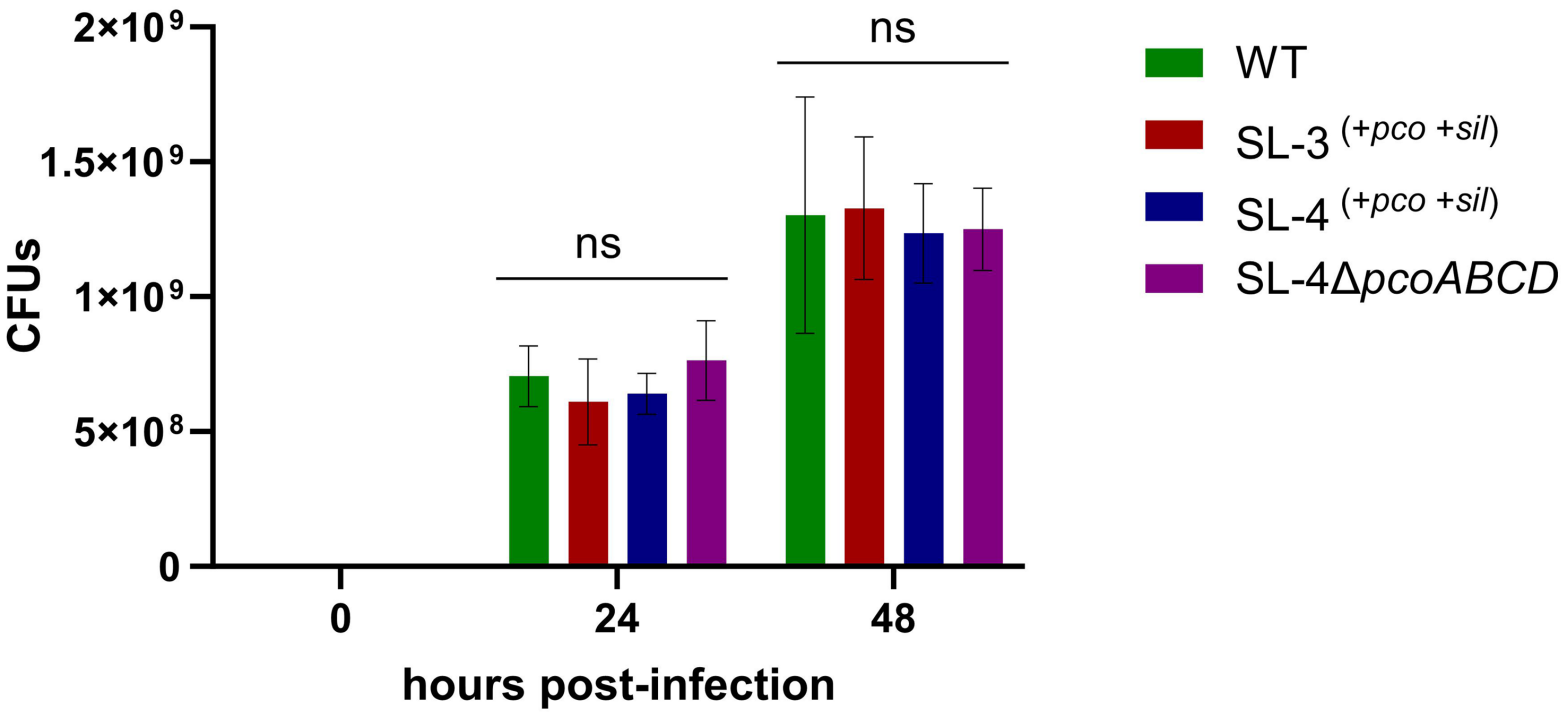
Loss of *pcoABCD* genes does not impact replication in RAW 264.7 macrophages. RAW 264.7 macrophages were infected with *S. enterica* (WT), SL-3, SL-4, and SL-4Δ*pco* at MOI = 1 for 1 h in DMEM supplemented with 10% FBS. At the indicated time points, cells were lysed and plated onto LB agar medium and CFUs were enumerated. Data shown indicate the mean CFU with standard error of two independent experiments with three biological replicates. The *p-*values were calculated by two-way ANOVA (ns, *p* > 0.05).

As an intracellular pathogen, *S. enterica* encounters high levels of copper in macrophages, specifically in phagolysosome, which is employed by host immune response to kill intracellular pathogens (Wagner et al., 2006), we assessed whether the deletion the of the *pco* genes cluster has an impact on replication during macrophage infection. We hypothesize that *pco* genes aid in the ability of SL-4 to overcome copper toxicity in phagolysosome, which in turn enhances survival in macrophages. The survival of SL-4Δ*pco*, SL-4, and wild-type *S. enterica* was examined in RAW264.7 macrophages, in which macrophages were infected with these strains at an MOI of 1. The infection experiment revealed that deletion of *pcoABCD* genes had no significant reduction in replication within RAW264.7 macrophages up to 72 h post-infection (Figure 5). These results indicate that *S. enterica* uses another mechanism to evade the macrophage intracellular killing.

## Discussion

In this study, we have clearly demonstrated that the acquired *pco* cluster functions in copper tolerance in non-typhoidal *S. enterica*, specifically under anaerobic conditions. Our *in silico* analysis reveals that *pco* and *sil* clusters are found in the chromosome (in SL-3) or on an IncFIB conjugative plasmid (in SL-4) and are not associated with AMR genes. *Pco* and *sil* clusters are mainly located on IncH-type plasmids and are co-localized with AMR genes (Li et al., 2021; Billman-Jacobe et al., 2018). To our knowledge, the integration of these clusters on IncFIB plasmid and the lack of association with AMR genes have not been reported previously. Our results show that a mutant lacking *pcoABCD* genes is sensitive to high levels of copper only under anaerobic condition, and that the *sil* cluster was not able to rescue the *pco* mutant. These results are consistent with the conversion of the non-toxic form of copper, Cu (II), to the toxic form, Cu (I) in anoxic environments (Beswick et al., 1976). Complementation of the SL-4Δ*pcoABCD* reversed growth phenotype to the same level of the parent strain. On the other hand, loss of these genes, had no impact on the capability of non-typhoidal *S. enterica* to replicate in macrophages. These data indicate that *S. enterica* may use the copper exporting P-type ATPase, GolT, as a surrogate for the *pco* cluster to overcome copper toxicity in macrophages (Pontel et al., 2014). The macrophage infection data is consistent with a previous study which showed that although deletion of *golT* resulted in accumulation of copper in the mutant, it did not impact replication in macrophages; however, survival in macrophages was compromised when cells were infected with the *golT* and *copA* double mutant (Osman et al., 2010).

**Figure 6:**
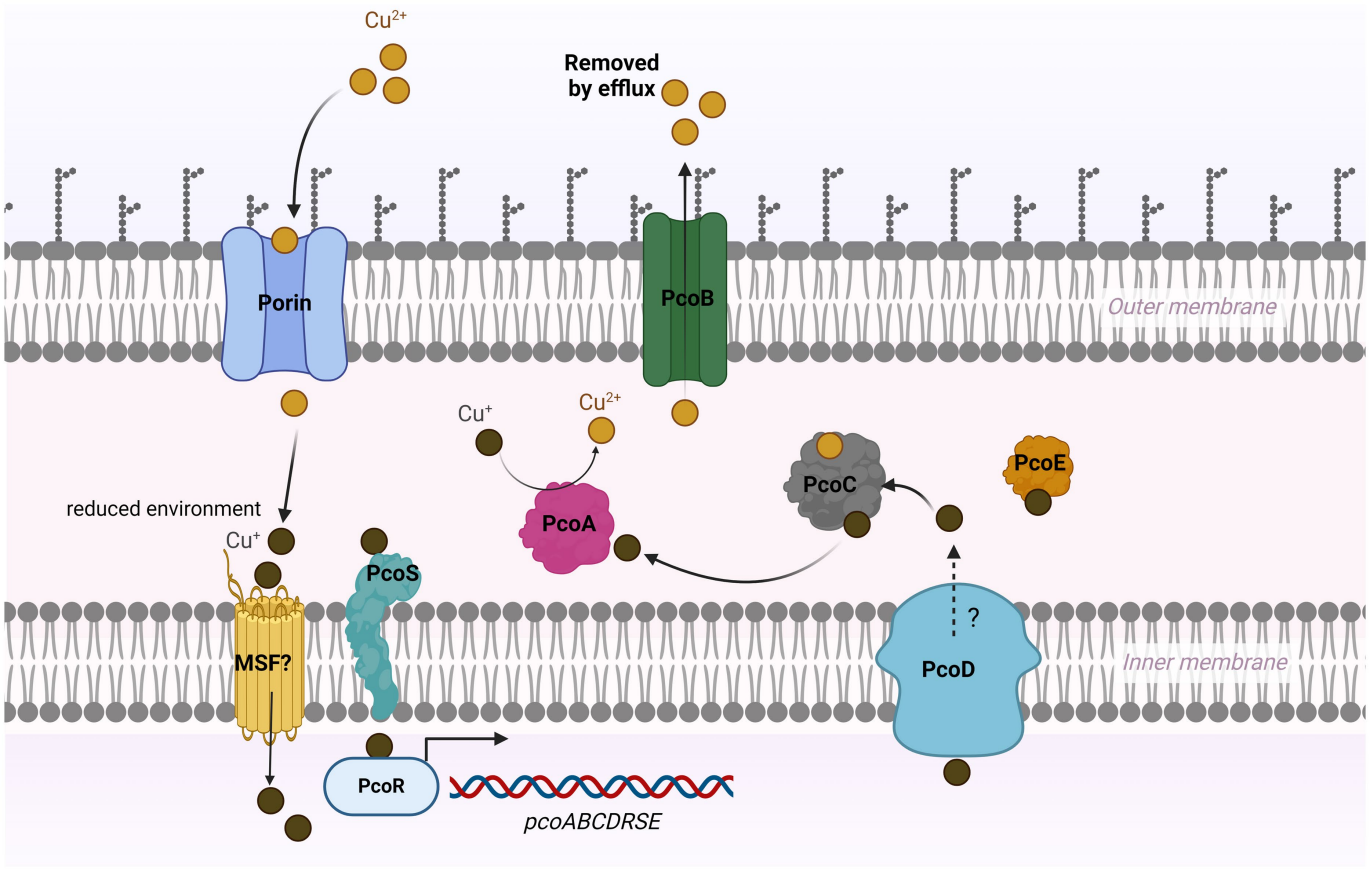
Model for copper resistance mediated by *pco* cluster in *Salmonella*. In a high-copper environment, copper enters bacterial cell via unknown mechanism that may involve outer membrane porin-like proteins and major facilitator super family (MSF). Cu (II) is converted to Cu (I) under reduced condition in the periplasm. The probable sensor protein PcoS may activate PcoR by phosphorylation, which then triggers the expression of the *pco* genes. PcoC binds both forms of Cu and transfers Cu (I) to the multicopper oxidase PcoA, where it is oxidized to the less reactive Cu (II). The Cu (I)-binding protein PcoE stores the excess (I) in the periplasm. PcoD may function as an efflux pump to extrude the intracellular Cu (I) to the periplasm. The outer membrane protein PcoB exports Cu (II) outside the cell.

The previous studies on the role of the *pco* cluster in copper tolerance in *S. enterica* are somewhat equivocal. Mourao et al., has reported that regardless of the presence or absence of *pcoA*-*D* genes along with *silA-E* genes in *S. enterica*, they are resistant to high levels of CuSO_4_ in anaerobic cultures (Mourao et al., 2016; Mourao et al., 2015). Both studies suggested that the *sil* cassette alone could manipulate copper homeostasis inside the cell. Our results show that *S.* Typhimurium (SL-27), which carries *sil* cluster only, was able to tolerate high levels of Cu (I) as shown in the growth curves and MIC data. These data indicate that this cluster can handle copper toxicity under anaerobic environment and are in accord with earlier studies (Mourao et al., 2016; Branchu et al., 2019; Mourao et al., 2015; Arai et al., 2019). Contrary to Aria’s and Branchu’s studies, where they did not detect MIC differences between the *pco* mutants and parent strains (Branchu et al., 2019; Arai et al., 2019), our data clearly show growth inhibition of the *pco* deletion mutant when grown in copper-supplemented media, which was restored by the complemented strain. The fact that *S.* Typhimurium encodes a multicopper oxidase (CueO), which protects from copper toxicity through the conversion of Cu (I) to Cu (II) (Achard et al., 2010), makes it possible that the copper resistance activity of the Pco proteins might be masked by CuO. Although the deletion of *silCBA* genes in Branchu’s study rendered the mutant sensitive to Cu (I), it was still able to tolerate up to 6–7 mM CuSO_4_ under anaerobic condition, and the MIC dropped dramatically below 2 mM upon deletion of both *sil* and *pco* cluster (Branchu et al., 2019). Indeed, their data support our hypothesis that *pco* cluster is required for full Cu (I) resistance when both clusters present together. Selection of copper resistance genes is attributed to the extensive use of copper as growth-promoter beyond the recommended concentrations in animal and poultry diet. Consistent with the level of copper (approximately 1–5 mM) detected in chickens manure fed on copper-supplemented diet (Korish and Attia, 2020), the copper-resistant strains were able to tolerate up to 7 mM, whereas the *pco* deletion mutant failed to grow in the presence of 1 mM CuSO_4_ under anaerobic conditions. Expression of *pcoABCDRSE* genes from the plasmid pPA173 in *E. coli* lacking the Cu (I)-translocating P-type ATPase (CopA) did not protect the Δ*copA* from copper toxicity but rescued the parent strain grown under the same condition (Lee et al., 2002). This study suggests that Pco proteins maintain copper homeostasis at the periplasmic level, and that CopA is required to further export excess copper across the outer membrane (Lee et al., 2002). Additionally, transformation of *E. coli* with plasmid pRJ1004, which contains the *pco* cluster, resulted in reduced accumulation of Cu (II) during initial exposure to ^64^Cu (II); however, ironically, the same strain accumulated more Cu (II) during the stationary phase, which may suggest that some of the Pco proteins transport Cu (II) across the inner membrane to the periplasmic space (Brown et al., 1995). In accordance with its role in *E. coli*, our seven-day growth curve results indicated that the *pco* cluster protects *S. enterica* from copper toxicity, but it remains unclear if copper is accumulated in the periplasmic space or is pumped across the outer membrane through PcoB. Interestingly, genome mining revealed that SL-3 and SL-4 harbor a gene that encodes a copper-transporting ATPase protein, which has homology to CopA that may act as an outer membrane efflux pump. Because we did not create a double mutation of *pcoABCD* and *copA*, we still do not know if *copA* is required for full copper tolerance in *S. enterica*. Thus, based on our data, we suggest that the Pco proteins function as a Cu (I) efflux system. In accordance with previous studies (Mourao et al., 2016; Mourao et al., 2015; Medardus et al., 2014), our data demonstrate that the loss of *pcoABCD* genes did not result in growth retardation in an aerobic environment, which is ascribed to the absence of the toxic cuprous form, Cu (I), in this condition. The copper resistance mechanism of the *pco* cluster stems from its ability to detoxify Cu (I) and extrude Cu (II) outside the bacterial cell (Zimmermann et al., 2012; Lawaree et al., 2016) as shown in our model (Figure 6).

The widespread usage of heavy metals potentially induces a selection pressure to allow pathogens thrive in hostile environment (Vats et al., 2022). Notably, copper has been widely used in food processing plants as a bactericide (Borkow and Gabbay, 2005), which may help explain the increased prevalence of the acquired copper-resistance genes in *Enterobacteriaceae* (Li et al., 2021; Mustafa et al., 2021).

Overall, this study highlights the role of the *pco* cluster in copper tolerance in non-typhoidal *S. enterica*.

## Data Availability

The original contributions presented in the study are included in the article/Supplementary material, further inquiries can be directed to the corresponding author/s.

## Footnotes

^1^http://galaxyproject.org
