# Supplementary material for: The acquired pco gene cluster in Salmonella enterica mediates resistance to copper
Source: Front Microbiol. 2024 Sep 3;15:1454763. doi: 10.3389/fmicb.2024.1454763 (PMC11406079; doi:10.3389/fmicb.2024.1454763)
Supplement: Supplementary file 1 [file Data_Sheet_1.PDF]

Supplementary information

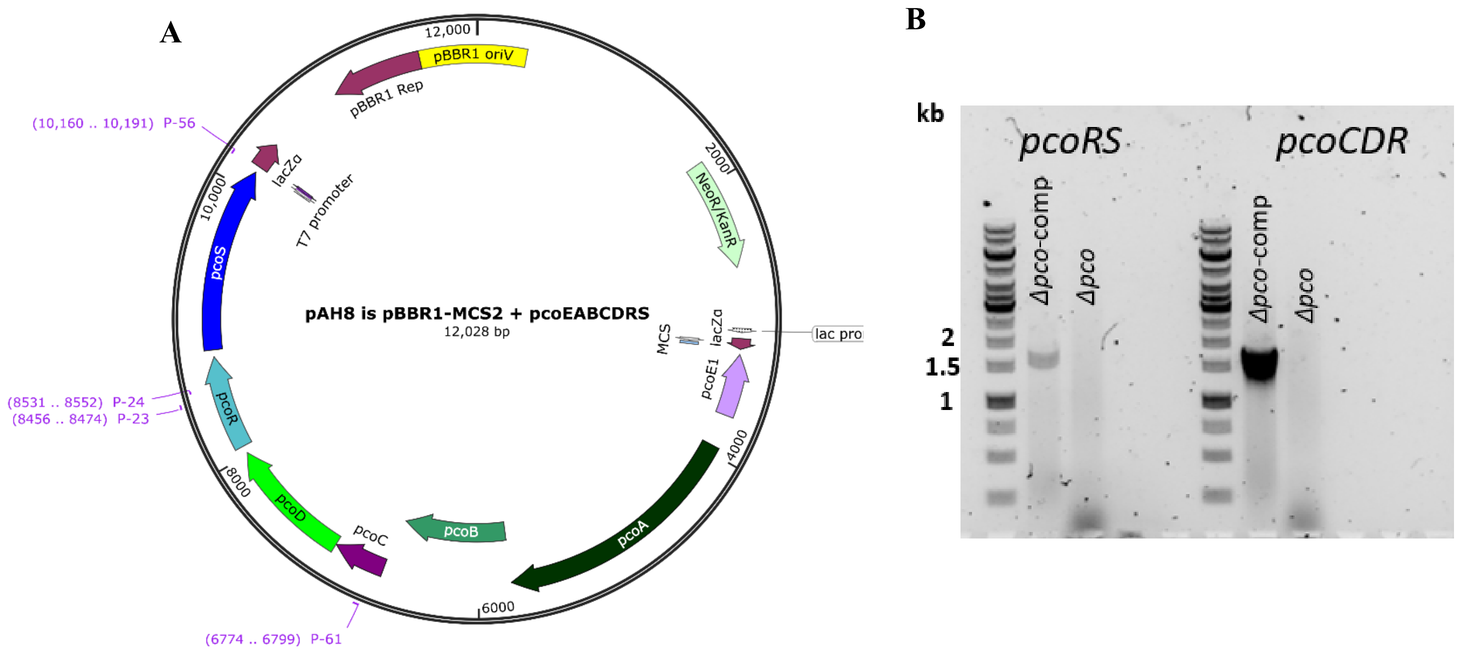

SI. 1. Confirmation of the complemented SL4*Δpco* strain

(A) Map for the plasmid pAH8 (generated by SnapGene 7) that was used to complement SL4Δ*pcoABCD*. The plasmid is a derivative of pBBR1-MCS2 and encodes *pcoEABCDRS* genes. **(B)** PCR screen for the insertion of *pco* cluster in the plasmid using primer pairs: P23/P56 and P61/P24. The primers' locations shown on the map. The expected product for screening of *pcoRS* and *pcoCDR* genes are 1.7 and 1.8 kb, respectively.

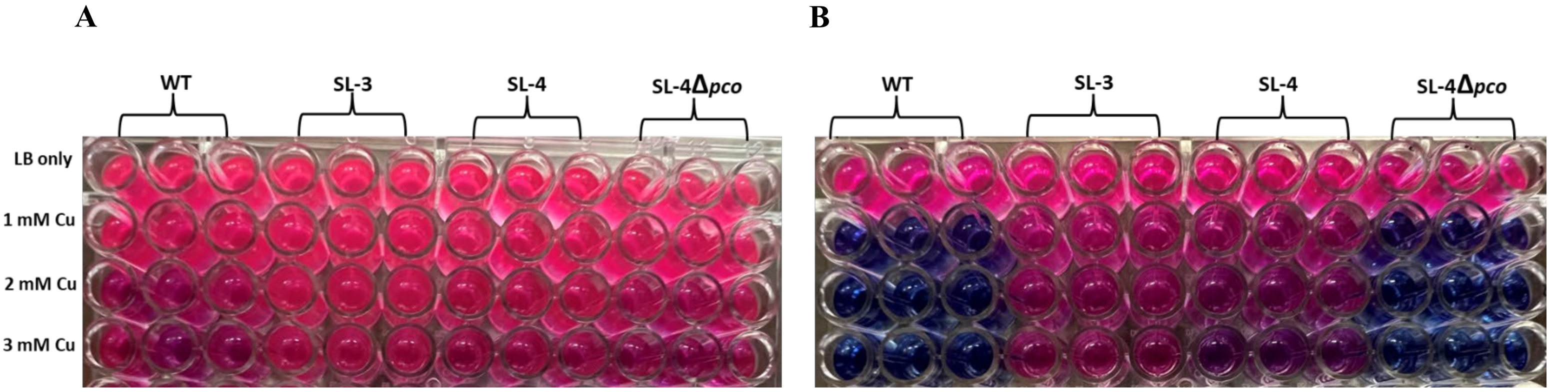

SI. 2. AlamarBlue assays for WT *S. enterica*, SL-3, SL-4, and SL4Δ*pco* cultures with the indicated concentrations of $CuSO_4$. Cultures were incubated aerobically **(A)** or anaerobically **(B)**. Growth is indicated by conversion of the indicator dye from blue to pink. The experiment was repeated three times.
